# Lipopolysaccharide infusion enhances dynamic cerebral autoregulation without affecting cerebral oxygen vasoreactivity in healthy volunteers

**DOI:** 10.1186/cc13062

**Published:** 2013-10-16

**Authors:** Ronan MG Berg, Ronni R Plovsing, Kevin A Evans, Claus B Christiansen, Damian M Bailey, Niels-Henrik Holstein-Rathlou, Kirsten Møller

**Affiliations:** 1 Centre of Inflammation and Metabolism, Department of Infectious Diseases, section M7641, University Hospital Rigshospitalet, Blegdamsvej 9, DK-2100, Copenhagen Ø, Denmark; 2 Renal and Vascular Research Section, Department of Biomedical Sciences, Faculty of Health Sciences, University of Copenhagen, Copenhagen N, Denmark; 3 Intensive Care Unit 4131, University Hospital Rigshospitalet, Copenhagen Ø, Denmark; 4 Neurovascular Research Laboratory, Faculty of Health, Science and Sport, University of Glamorgan, South Wales CF374AT, UK; 5 Neurointensive Care Unit 2093, Department of Neuroanaesthesiology, University Hospital Rigshospitalet, Copenhagen Ø, Denmark

## Abstract

**Introduction:**

Sepsis may be associated with disturbances in cerebral oxygen transport and cerebral haemodynamic function, thus rendering the brain particularly susceptible to hypoxia. The purpose of this study was to assess the impact of isocapnic hypoxia and hyperoxia on dynamic cerebral autoregulation in a human-experimental model of the systemic inflammatory response during the early stages of sepsis.

**Methods:**

A total of ten healthy volunteers were exposed to acute isocapnic inspiratory hyperoxia (F_I_O_2_ = 40%) and hypoxia (F_I_O_2_ = 12%) before and after a 4-hour lipopolysaccharide (LPS) infusion (2 ng kg^-1^). Middle cerebral artery blood follow velocity was assessed using transcranial Doppler ultrasound, and dynamic autoregulation was evaluated by transfer function analysis.

**Results:**

Transfer function analysis revealed an increase in the phase difference between mean arterial blood pressure and middle cerebral artery blood flow velocity in the low frequency range (0.07–0.20 Hz) after LPS (*P*<0.01). In contrast, there were no effects of either isocapnic hyperoxia or hypoxia on dynamic autoregulation, and the cerebral oxygen vasoreactivity to both hyperoxia and hypoxia was unaffected by LPS.

**Conclusions:**

The observed increase in phase suggests that dynamic cerebral autoregulation is enhanced after LPS infusion and resistant to any effects of acute hypoxia; this may protect the brain from ischaemia and/or blood–brain barrier damage during the early stages of sepsis.

## Introduction

Dynamic cerebral autoregulation functions to maintain cerebral oxygen delivery by dampening the effects of acute surges in cerebral perfusion pressure on cerebral blood flow (CBF) through cerebral arteriolar vasodilatation and vasoconstriction as mean arterial blood pressure (MAP) decreases and increases, respectively [1,2]. It functions in concert with cerebral oxygen vasoreactivity (COVR), which maintains oxygen delivery by means of cerebral arteriolar vasoconstriction or vasodilatation following increases and decreases in PaO_2_, respectively, to optimise cerebral tissue oxygenation [3]. These homeostatic mechanisms are important to support the metabolic demands of neuronal activity and the need to protect brain tissue from the potentially damaging effects of hypo- and hyperperfusion [1,2].

Dynamic cerebral autoregulation has previously been found to operate with a prolonged response time in critically ill patients with sepsis [4-7]. This may contribute to symptoms of encephalopathy as well as long-term cognitive dysfunction, both of which are commonly encountered in this patient group [8,9], by exposing the brain to intermittent ischaemia and blood–brain barrier damage during acute decreases and increases in MAP, respectively. In contrast, dynamic cerebral autoregulation has been found to be improved after lipopolysaccharide (LPS) administration in healthy volunteers [4,10], a human-experimental model of the systemic inflammatory response during early sepsis [11]. This discrepancy suggests that dynamic autoregulation is maintained during the early stages of sepsis but then becomes 'indolent’ during the clinical course of disease, in the sense that the magnitude of the cerebrovascular response to a given change in MAP is preserved, while the response time of the cerebrovasculature becomes progressively prolonged [4]. The changes in dynamic autoregulation may be related to PaCO_2_ levels, in that the hyperventilatory response induced by LPS infusion may improve dynamic autoregulation [10], while the prolonged response time of the cerebral circulation in patients is associated with higher PaCO_2_ levels [4,7]. Furthermore, the fever response may contribute to the improvement in dynamic autoregulatory performance observed after LPS infusion, since passive hyperthermia has previously been shown to shorten the response time of the cerebral circulation [12,13]. An additional contributory factor to the slower dynamic autoregulatory responses in patients may be the presence of hypoxia, which is common in sepsis [4], since even brief bouts (minutes) of inspiratory hypoxia may impair dynamic autoregulation [14]. Furthermore, the presence of systemic inflammation may render the cerebral circulation more susceptible to hypoxia by facilitating the formation of reactive oxygen-nitrogen species [15]. As of now it is unknown whether systemic inflammation enhances the effects of acute hypoxia on dynamic cerebral autoregulation.

In the present study, we examined the effects of acute inspiratory hyperoxia and hypoxia on dynamic cerebral autoregulation during an LPS-induced systemic inflammatory response in healthy volunteers. Since changes in PaCO_2_ induced by hypoxia and hyperoxia may in themselves affect cerebral autoregulation, an attempt was made to keep the volunteers isocapnic by continually monitoring and adjusting end-tidal CO_2_ (P_ET_CO_2_) during the cerebral autoregulatory assessments. We hypothesised that: 1) dynamic autoregulation would be enhanced after LPS during normoxia; 2) dynamic cerebral autoregulation would be impaired during acute hypoxia at baseline; and 3) the impact of hypoxia on dynamic autoregulation would be augmented after LPS.

## Material and methods

The study was approved by the Scientific Ethical Committee of Copenhagen and Frederiksberg Municipalities, Denmark (file number H-2-2010-04), and was performed in accordance with the Declaration of Helsinki [16].

### Participants

Ten healthy non-sedentary male volunteers, 23 (mean) ± 2 (SD) years old, were included in the study after having provided oral and written informed consent. All volunteers had an unremarkable medical history, with no signs of infection within four weeks before the trial day, and none took any regular medication. Prior to inclusion, all volunteers underwent a thorough physical examination, a 12-lead electrocardiogram (ECG) was obtained, and standard laboratory tests were performed. All tests were normal.

On the study day, volunteers reported to the ICU at 7:00 am following a 12- hour overnight fast. They were placed in bed and catheterised with an antecubital catheter (for LPS infusion), and an arterial line was inserted in the left radial artery following local anaesthesia (lidocaine, 20 mg.ml^-1^). The volunteers then rested for 45 minutes while remaining supine before measurements were started. Heart rate (via a three-lead ECG), invasive blood pressure and arterial oxygen saturation (by pulse oximetry) were continuously monitored, and medically qualified personnel were present at all times. External stimuli were minimised during all measurements. Volunteers were allowed to drink tap water *ad libitum* during the study day. After completion of all interventions, volunteers were monitored until complete alleviation of symptoms and normalisation of all vital parameters, and they were discharged following a light meal and removal of catheters.

### Lipopolysaccharide infusion

Volunteers underwent a four-hour continuous intravenous infusion of purified *Escherichia coli* LPS (infusion rate, 0.5 ng.kg^-1^. hour^-1^; Batch G2 B274, US Pharmacopeial Convention, Rockville, MD, USA). In this model, volunteer symptoms and plasma levels of the pro-inflammatory cytokine tumour necrosis factor (TNF)-α reach maximal values at approximately one hour after cessation of the infusion [17]. Rectal temperature was measured at baseline, hourly during the LPS infusion, and at six hours, that is, two hours after cessation of the infusion. Volunteer symptoms were evaluated at these time points by means of a modified visual analogue scale, as previously described [4]. Briefly, six individual symptom scores, encompassing malaise, shivering, nausea, headache, myalgia and dizziness, were added together to yield a total symptom score with a minimal value of 6, implying no symptoms at all, and a maximal value of 60, indicating that all symptoms are the worst imaginable. Symptoms of encephalopathy were not systematically assessed during the study.

### Inspiratory hyperoxia and hypoxia

Prior to (baseline) and immediately after (at four hours) LPS infusion, three interventions were conducted using a closed system (Ambu 'E’ valve) with a tight-fitting mask: 20 minutes of normoxia (21% O_2_; 79% N_2_), 20 minutes of inspiratory hyperoxia (40% O_2_; 60% N_2_), and 20 minutes of inspiratory hypoxia (12% O_2_; 88% N_2_). Normoxia was the first intervention both before and after LPS in all volunteers, and this was immediately followed by hyperoxia or hypoxia in a volunteer-blinded block-randomised fashion; hence, hyperoxia was conducted before hypoxia in five subjects and after hypoxia in the remaining five. The hyperoxic and hypoxic interventions were divided by a normoxic washout period of 20 minutes, so that the three interventions lasted for a total of approximately 80 minutes. The inspired gases were continually added to a 3-litre polychloroprene anaesthesia breathing bag (Nolato Medical, Torekov, Sweden) with approximately 30 ml water at room temperature for vaporisation; the breathing bag was connected to the face mask through a flexible low-resistance system, and the total instrumental dead space was less than 300 ml. To avoid the contaminating effects of hypocapnia-mediated cerebral vasoconstriction, all interventions were conducted in isocapnia by continuously monitoring P_ET_CO_2_ using a capnograph (M3015A CO_2_ MMS Module, Philips Medical Systems, Böblingen, Germany) attached to the face mask, and adding CO_2_ to the inspired air *ad hoc*, aiming at a P_ET_CO_2_ between 5.5 and 5.8 kPa.

### Transcranial Doppler ultrasound

A 2 MHz pulsed transcranial Doppler ultrasound (TCD) system (Ez-Dop and Doppler-Box, Compumedics DWL GmbH, Singen, Germany) was used to measure backscattered Doppler signals continuously for the estimation of unilateral linear middle cerebral artery blood flow velocity (MCAv). Changes in MCAv were considered to reflect changes in CBF, assuming an unchanged calibre of the middle cerebral artery; TCD has previously been validated in this context during physiological changes in MAP and PaCO_2_[18-20]. Following a standardised search technique [21], the Doppler probe was fixed over the left transtemporal window using an adjustable silicone headband (provided by Compumedics DWL GmbH, Singen, Germany) to maintain the optimal angle of insonation during measurements. The TCD was detached during the four-hour LPS infusion and reattached and fixed before the interventions after LPS infusion.

The arterial line was attached to a pressure transducer (Invasive Blood Pressure Module M1006B, Philips Medical Systems, Böblingen, Germany), positioned at the level of the right atrium for the monitoring and recording of beat-to-beat arterial blood pressure. The subject remained supine with slight head elevation at approximately 20° during all measurements. Invasive arterial blood pressure, TCD waveforms and respiratory rate were sampled continuously at 1 kHz using an analogue-to-digital converter (PowerLab 16/30™, ADInstruments Ltd, Oxford, UK) and recorded in LabChart version 7 (ADInstruments Ltd, Oxford, UK) on an offline personal computer. The presented values for MAP, heart rate, MCAv and respiratory rate are the mean values recorded over the four minute period immediately preceding the given time point.

### Dynamic cerebral autoregulation

Dynamic autoregulation was evaluated using transfer function analysis [22], which assesses the impact of spontaneous oscillations in MAP on MCAv. To ensure that steady state had been attained during the hyperoxic and hypoxic interventions, recordings of invasive arterial blood pressure and TCD waveforms from the last 10 minutes of the interventions were used for spectral analysis after re-sampling at 10 Hz. A 1,200-point Fourier transformation, with a 60 second overlap between sequential segments for Welch spectral estimation and multiplication by the Hanning window to minimise spectral leakage, was performed in MatLab version R2011a (MathWorks, Natick, MD, USA). Data were analysed in the very low (0.02 to 0.07 Hz), low (0.07 to 0.20 Hz) and high (0.20 to 0.30 Hz) frequency ranges. Transfer gain, normalised gain, MAP-to-MCAv phase difference and coherence were subsequently calculated [22,23], and data from the low frequency range were specifically used as the basis for assessing dynamic autoregulatory performance, since contamination of the MAP fluctuations by respiration is minimal within this frequency range [22,24]; to ensure valid transfer gain, normalised gain, and MAP-to-MCAv phase difference estimates, only values where the corresponding coherence was ≥0.4 were used [2]. Accordingly, a decrease in transfer gain, normalised gain, and/or an increased MAP-to-MCAv phase difference would be interpreted as evidence for enhanced dynamic autoregulation and *vice versa*[22,23].

### Cerebral oxygen vasoreactivity

The % change in MCAv per kPa change in PaO_2_ was used as an estimate of the COVR [3]. Due to the nonlinear relationship between PaO_2_ and CBF, COVR was calculated separately for hyperoxia and hypoxia. A F_I_O_2_ of 40% was chosen for the induction of hyperoxia, because this would expectedly increase PaO_2_ above 13.3 kPa, above which the relationship between PaO_2_ and CBF becomes asymptotic, so that further changes in CBF are minimal [3]. A F_I_O_2_ of 12% was chosen for the induction of hypoxia because this would expectedly decrease PaO2 to approximately 7.9 kPa, below which the relationship between PaO_2_ and CBF becomes hyperbolic with large changes in CBF upon relatively small changes in PaO_2_[3,25]. The COVR was calculated using the average MCAv values obtained during the last four minutes of the normoxic, hyperoxic and hypoxic interventions.

### Laboratory analyses

Blood samples for white blood cell counts were obtained from the arterial catheter at baseline, hourly during the LPS infusion, and two hours after cessation of the infusion (at six hours) and were determined by an automated analyser (Sysmex XE-2100, Sysmex Europe GmbH, Hamburg, Germany).

Blood samples for the determination of plasma TNF-α and IL-6 concentrations were obtained at baseline and after LPS, at the end of the normoxic, hyperoxic and hypoxic interventions, respectively. Plasma was obtained by centrifuging whole blood in ethylenediaminetetraacetic acid (EDTA)-containing tubes at 3,000 rpm at 4°C for 10 minutes and kept at -80°C until analysis. TNF-α and IL-6 were measured in duplicate by means of electrochemiluminescent detection on a multiplex immunoassay using a SECTOR Imager 2400 (Meso Scale Diagnostics, Gaithersburg, MD, USA), and mean concentrations were calculated. The interassay coefficient of variation at the high and low end of the standard curve was assessed by using two internal controls (human plasma) and was below 12% at a known concentration of 2.4 pg.ml^-1^ and below 8% at a known concentration of 10,000 pg.ml^-1^ for both cytokines.

Arterial blood gases were obtained from the arterial catheter at baseline, hourly during the LPS infusion, and at the end of the normoxic, hyperoxic and hypoxic interventions, both at baseline and after LPS. Arterial oxygen tension (PaO_2_), oxygen saturation (SaO_2_), PaCO_2_, pH, HCO_3_^-^, base excess and haemoglobin were subsequently determined on a blood gas analyser (ABL 725, Radiometer, Brønshøj, Denmark).

The arterial blood content of oxygen (CaO_2_) was calculated as

$$\begin{array}{ll} \;{\mathrm{CaO}}_{2}\left({\mathrm{mmol}\;L}^{-1}\right)= & {\mathrm{SaO}}_{2}\left(\mathrm{fraction}\right)\\ & \times \mathrm{haemoglobin}\;\left({\mathrm{mmol}\;L}^{-1}\right)\\ & +{\mathrm{PaO}}_{2}\left(\mathrm{kPa}\right)\\ & \times 0.01{\;\mathrm{mmol}\;L}^{-1}{\mathrm{kPa}}^{-1} \end{array}$$

### Statistics

A linear mixed model with a covariance structure fitted for non-equidistant data points was used to analyse the effects of LPS on white blood cell counts, symptom scores, as well as cardiovascular and respiratory parameters over time. If an overall effect was identified (LPS × time interaction), the estimated individual differences from baseline were evaluated using the Tukey-Kramer adjustment for multiple comparisons. Standard residual diagnostics were used for model control; all data showed variance homogeneity and normal distribution of residuals. Since the majority of cerebral haemodynamic data and cytokines did not follow a normal distribution, as evaluated by normality plots and Shapiro-Wilk’s test for normality, Wilcoxon’s signed-rank test was used for paired comparisons between normoxia, hyperoxia, and hypoxia before and after LPS, and *P*-values were adjusted according to Holm’s sequential Bonferroni correction. Unless otherwise stated, data are presented as median with the corresponding interquartile range. Statistical significance was attained at *P*<0.05, and all statistical analyses were performed by SAS statistical software version 9.2 (SAS Institute Inc., Cary, NC, USA).

## Results

### Systemic inflammatory response

**Figure 1:**
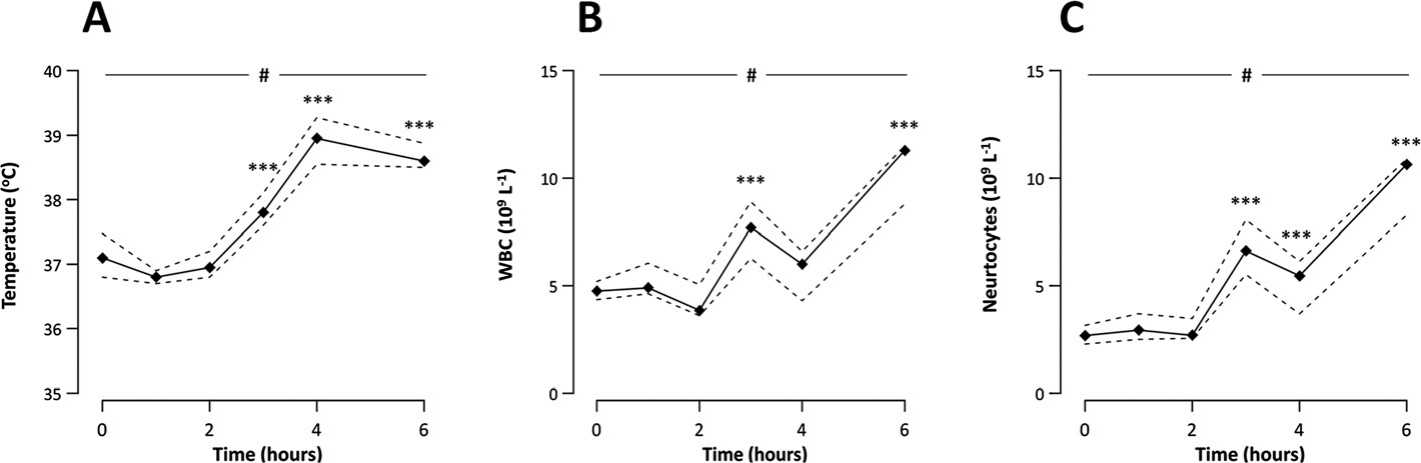
**Temperature and white blood cell response.** A blood sample was obtained from a radial artery catheter at baseline and hourly during a four-hour lipopolysaccharide (LPS) infusion, as well as two hours after cessation of the infusion, in healthy volunteers (n = 10). **A**: Temperature; **B**: White blood cell count (WBC); **C**: Neutrocyte count. Data are presented as median (black line) and interquartile range (dashed lines). LPS x time interaction, # *P*<0.001. Different from baseline, *** *P*<0.001.

LPS infusion was associated with fever (Figure 1A) and increases in white blood cell count with neutrocytosis (Figure 1B-C), as well as increases in symptom scores. The individual symptoms and the total symptom score are outlined in an additional file [see Additional file 1]. This was accompanied by a progressive increase in heart rate and respiratory rate (both *P*<0.001); heart rate increased from 53 (51 to 56) to 93 (85 to 104) beats.min^-1^ while respiratory rate increased from 11 (9 to 13) to 19 (16 to 22) breaths.min^-1^, both peaking at four hours (both *P*<0.001 compared to baseline). The corresponding PaCO_2_ decreased during LPS infusion (*P*<0.001), from 5.7 (5.6 to 5.8) kPa at baseline to 4.8 (4.6 to 4.9) kPa at four hours (*P*<0.001), and pH subsequently increased (*P*<0.001) from 7.39 (7.38 to 7.44) at baseline to 7.44 (7.43 to 7.44) at four hours (*P*<0.05). MAP was 88 (84 to 90) mmHg at baseline and 87 (81 to 90) mmHg at four hours and was unaffected by LPS (*P* = 0.59).

### Inspiratory hyperoxia and hypoxia

PaO_2_, SaO_2_ and CaO_2_ were affected to the same extent by inspiratory hyperoxia and hypoxia at baseline and after LPS (Table 1). At baseline, the respiratory rate was 11 (9 to 13) breaths . min^-1^ during hyperoxia and 12 (11 to 13) during hypoxia; after LPS, it was 13 (12 to 16) breaths.min^-1^ during hyperoxia, and 15 (13 to 20) during hypoxia with no differences between interventions. P_ET_CO_2_ was kept constant during the interventions, and apart from a small decrease during inspiratory hypoxia after LPS, values of P_ET_CO_2_ were similar at baseline and after LPS; although the corresponding PaCO_2_ levels were consistently lower after LPS, none of the subjects were hypocapnic (PaCO_2_ <4.5 kPa) during the interventions (Table 1).

**Table 1:** **Arterial blood gas parameters and end-tidal CO**_**2 **_**(P**_**ET**_**CO**_**2**_**)**

	**Baseline**			**LPS**		
	**Normoxia**	**Hyperoxia**	**Hypoxia**	**Normoxia**	**Hyperoxia**	**Hypoxia**
PaO_2_ (kPa)	13.2	28.5**	7.5**	11.6†	26.7**	7.1**
	(12.8–14.3)	(27.4–29.1)	(6.0–8.2)	(11.0–13.1)	(26.4–27.0)	(6.6–7.3)
SaO_2_ (%)	98	100**	90**	98	100**	89**
	(98–99)	(100–100)	(82–92)	(98–98)	(100–100)	(85–91)
CaO_2_ (mM)	8.4	8.7*	7.5**	8.3	8.7**	7.4**
	(8.1–8.9)	(8.6–9.0)	(7.2–7.8)	(8.0–8.8)	(8.3–9.1)	(7.1–8.3)
PaCO_2_ (kPa)	5.7	5.6	5.7	5.0††	5.0††	4.8††
	(5.6–5.8)	(5.2–6.0)	(5.5–5.8)	(5.0–5.1)	(4.8–5.0)	(4.7–5.1)
pH (units)	7.39	7.40	7.41*	7.43†	7.43	7.45*††
	(7.38–7.41)	(7.40–7.42)	(7.39–7.42)	(7.42–7.44)	(7.41–7.45)	(7.42–7.47)
HCO_3_^-^ (mM)	25.0	25.1	25.0	25.3	25.9	25.5
	(24.4–26.6)	(23.9–26.4)	(24.5–26.2)	(24.8–26.0)	(24.7–26.7)	(24.2–26.0)
Base excess (mM)	0.9	1.0	1.6	1	1	0.9†
	(0.2–2.7)	([-0.4]–2.4)	(0.7–2.7)	(0.2–2.3)	(0.1–2.1)	(0–2.4)
P_ET_CO_2_ (kPa)	5.4	5.2	5.2	5.3	5.2	5.1*
	(5.1–5.4)	(5.2–5.4)	(5.1–5.3)	(5.2–5.4)	(5.1–5.3)	(5.1–5.2)

A blood sample was obtained from the radial artery catheter during three interventions, normoxia (F_I_O_2_ = 21 %), hyperoxia (F_I_O_2_ = 40%) and hypoxia (F_I_O_2_ = 12%), at baseline, and after a four-hour lipopolysaccharide (LPS) infusion in healthy volunteers (n = 10). CaO_2_: arterial oxygen content. Data are presented as median (interquartile range). Different from normoxia in the same condition (baseline/LPS), *P<0.05, **P<0.01. Different from the same intervention (normoxia/hyperoxia/hypoxia) at baseline, †*P*<0.01, ††*P*<0.001.

**Figure 2:**
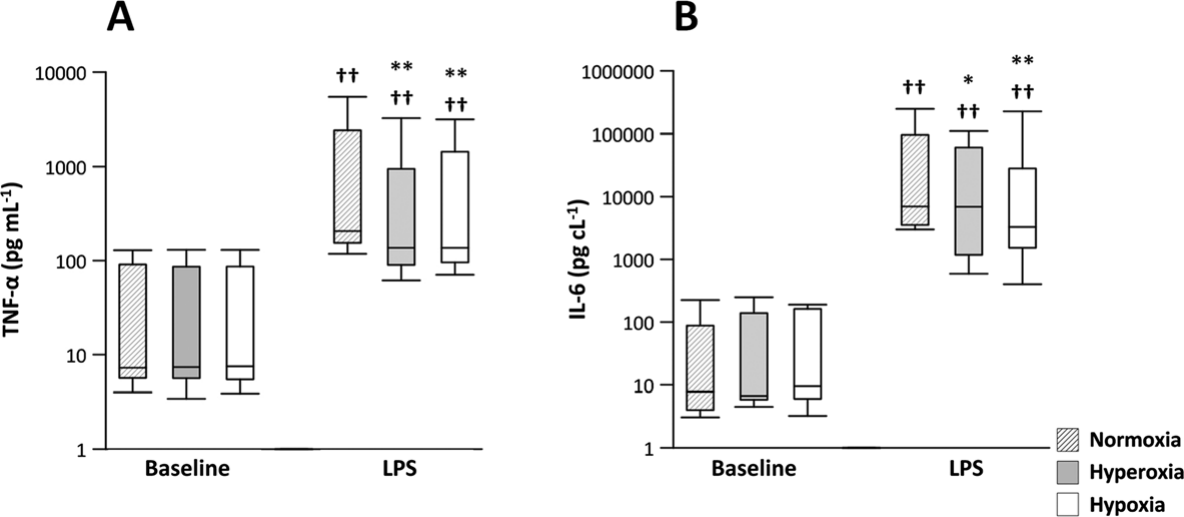
**Cytokine response.** A blood sample was obtained from the radial artery catheter during three interventions, normoxia (F_I_O_2_ = 21%), hyperoxia (F_I_O_2_ = 40%) and hypoxia (F_I_O_2_ = 12%), at baseline, and after a four-hour lipopolysaccharide (LPS) infusion in healthy volunteers (n = 10). **A**: Plasma TNF-α; **B**: Plasma IL-6. Data are presented as median (black line), interquartile range (IQR, box) and total range (whiskers). Different from normoxia in the same condition (baseline/LPS), ** *P*<0.01. Different from the same intervention (normoxia/hyperoxia/hypoxia) at baseline, †† *P*<0.001.

TNF-α and IL-6 levels were similar during normoxia, hyperoxia and hypoxia at baseline; they increased markedly after LPS, and both were higher during normoxia than during hyperoxia and hypoxia (Figure 2A-B). MAP was affected neither by hyperoxia nor by hypoxia before or after LPS (NS, data not shown).

### Dynamic cerebral autoregulation

Similar MCAv values were obtained during normoxia at baseline and after LPS (72 (67 to 76) at baseline versus 69 (64 to 77) cm.sec^-1^ after LPS; *P* = 0.97). In one subject, coherence in the low frequency range decreased to 0.28 during hypoxia at baseline; this subject was therefore excluded for the assessment of dynamic cerebral autoregulation. The MAP-to-MCAv phase difference increased after LPS, whereas transfer gain and normalised gain were similar to baseline values (Table 2). Transfer gain, normalised gain, and the MAP-to-MCAv phase difference were unaffected by hyperoxia and hypoxia, both before and after LPS (Table 2). Similar results were obtained when the data points, in which the respiratory rate was within the low frequency range, were omitted from the analysis. Results of the transfer function analysis in all 10 subjects in the very low, low and high frequency ranges are presented in an additional file [see Additional file 2].

**Table 2:** Dynamic cerebral autoregulation

	**Baseline**			**LPS**		
	**Normoxia**	**Hyperoxia**	**Hypoxia**	**Normoxia**	**Hyperoxia**	**Hypoxia**
MAP_sp_ (mmHg^2^)	4.6	4.6	8.7	3.9	7.8	9.0
	(4.3–6.4)	(4.2–6.7)	(4.6–14.5)	(1.9–10.8)	(4.1–8.3)	(2.2–11.0)
MCAv_sp_ (cm^2^ sec^-2^)	9.2	8.9	10.6	5.6	11.7	13.1
	(5.6–10.1)	(6.0–11.7)	(7.6–19.4)	(2.4–15.3)	(8.3–14.1)	(5.7–23.4)
Gain (cm mmHg^-1^ sec^-1^)	1.26	1.34	1.28	1.20	1.24	1.29
	(1.03–1.37)	(1.08–1.38)	(1.15–1.35)	(1.10–1.24)	(1.00–1.28)	(1.17–1.33)
Normalised gain (units)	1.49	1.30	1.43	1.40	1.24	1.32
	(1.43–1.62)	(1.26–1.44)	(1.35–1.49)	(1.24–1.51)	(1.16–1.45)	(1.15–1.40)
Phase (radians)	0.58	0.70	0.47	0.82^a^	0.81^a^	0.91^a^
	(0.47–0.73)	(0.48–0.78)	(0.45–0.62)	(0.76–0.84)	(0.78–1.03)	(0.76–1.01)
Coherence (units)	0.87	0.90	0.91	0.79	0.86	0.88
	(0.86–0.88)	(0.86–0.93)	(0.89–0.93)	(0.71–0.85)	(0.83–0.86)	(0.69–0.94)

Dynamic cerebral autoregulation was tested during three interventions, normoxia (F_I_O_2_ = 21 %), hyperoxia (F_I_O_2_ = 40%) and hypoxia (F_I_O_2_ = 12%), at baseline, and after a four-hour lipopolysaccharide (LPS) infusion in healthy volunteers (n = 9). Data are presented as median (interquartile range, IQR). Values for the spectral power of mean arterial blood pressure (MAP_sp_) and middle cerebral artery blood flow velocity (MCAv_sp_), as well as transfer gain, the MAP-to-MCAv phase difference, and coherence are presented for the low frequency range (0.07 to 0.20 Hz). Only data for subjects with coherence ≥0.4 are presented. Different from the same intervention (normoxia/hyperoxia/hypoxia) at baseline, ^a^*P*<0.01.

### Cerebral oxygen vasoreactivity

At baseline, hyperoxia decreased MCAv from 74 (65 to 81) to 70 (59 to 73) cm.sec^-1^, while hypoxia increased MCAv from 67 (60 to 75) to 78 (68 to 88) cm.sec^-1^ (both *P*<0.01). After LPS, two subjects exhibited a paradoxical increase in MCAv during hyperoxia, but in the group as a whole, MCAv tended to decrease from 69 (64 to 73) to 63 (59 to 73) cm.sec^-1^ (*P* = 0.19). Hypoxia increased MCAv from 69 (61 to 77) to 78 (69 to 85) cm.sec^-1^ (*P*<0.01) after LPS.

There was no difference in COVR between baseline and LPS, neither to hyperoxia nor to hypoxia. At baseline, hyperoxia caused a 0.7 (0.3 to 1.2)% decrease in MCAv per kPa change in PaO_2_, whereas a 0.2 (0 to 0.6)% decrease in MCAv per kPa change in PaO_2_ was observed after LPS (baseline versus LPS, *P* = 0.50). Hypoxia caused a 1.1 (0.8 to 2.1)% increase in MCAv per kPa change in PaO_2_ at baseline; after LPS, hypoxia induced a 2.1 (1 to 4 to 4.5)% increase in MCAv per change in PaO_2_ (baseline versus LPS, *P* = 0.28). Similar findings were made when COVR calculations were based on CaO_2_ rather than PaO_2_ (data not shown).

## Discussion

In the present study we found that LPS infusion was associated with enhanced dynamic cerebral autoregulation in healthy volunteers, as indicated by an increased MAP-to-MCAv phase difference in the low frequency range. There was no effect of hypoxia on dynamic autoregulation, neither before nor after LPS, and the COVR to both hyperoxia and hypoxia was maintained.

LPS infusion induced a systemic inflammatory response similar to that encountered during the very early stages of sepsis, with fever, increased heart rate, tachypnoea and hyperventilation, leukocytosis, as well as elevated TNF-α and IL-6 levels [4,11]. We found that dynamic cerebral autoregulation was improved under these conditions, with unaffected transfer gain and normalised gain, but an increased MAP-to-MCAv phase difference. This implies that the magnitude of the cerebrovascular response to a given physiological change in MAP is similar, but that the cerebral circulation responds more rapidly to a given change.

The present findings are supported by two recent transfer function analysis-based studies that likewise found improved dynamic cerebral autoregulation after LPS administration [4,10]. The findings from these studies do differ somewhat from the data at hand, since the improvement in dynamic cerebral autoregulation involved both decreased transfer gain and an increased MAP-to-MCAv phase difference. The volunteers in these former studies were, however, poikilocapnic. Although we attempted to keep the healthy volunteers isocapnic by continually monitoring and adjusting P_ET_CO_2_ in the present study, LPS likewise induced a hyperventilatory response with lower PaCO_2_ levels after LPS than at baseline. This decrease in PaCO_2_ was, however, less pronounced than in a former study using the same four-hour LPS infusion [4]; hence, PaCO_2_ levels, transfer gain, and the MAP-to-MCAv phase differences were similar to the present study at baseline, but after LPS, PaCO_2_ levels decreased to 4.8 (4.6 to 5.0) kPa, and this was associated with lower transfer gain (0.87 (0.77 to 0.89) cm.mmHg^-1^.sec^-1^) and higher MAP-to-MCAv phase difference values (1.12 (1.10 to 1.31) radians) than observed in the present study. The potential impact of LPS-induced hyperventilation on the cerebral circulation was, therefore, likely dampened in the present study, and our findings may thus suggest that the changes in transfer gain and the MAP-to-MCAv phase difference observed in the former studies were facilitated by hypocapnia.

The increased MAP-to-MCAv phase difference, which remained even after alleviation of the PaCO_2_ decrease, may suggest that a relatively smaller change in PaCO_2_ is required to enhance the response time of the cerebral circulation (MAP-to-MCAv phase difference) than is required to improve its actual ability to dampen the impact of a given change in MAP on CBF (transfer gain and normalised gain). A possible additional contributory factor that may be of importance is the observed fever response. Accordingly, passive heating to core temperatures similar to those induced by LPS has been found to increase only the MAP-to-MCAv phase difference, whereas gain is unaffected [13]. The present findings thus support the notion that hyperventilation and fever may act in synergy to protect the cerebral circulation during the very early stages of sepsis [4].

We found no effects of either acute inspiratory hyperoxia or hypoxia on dynamic cerebral autoregulation at baseline or after LPS. It is well established that acute hyperoxia has no or minimal effects on dynamic autoregulation in healthy volunteers [14,26], and, based on our data, this is also not the case during an LPS-induced systemic inflammatory response. With regard to hypoxia, our findings disagree somewhat with former studies. Although acute inspiratory hypoxia with an F_I_O_2_ of 12% to 15% and a duration of 5 to 15 minutes has previously been found to be associated with unaltered, impaired or even improved dynamic cerebral autoregulation in healthy volunteers [26-28], these findings may have been biased by hypoxia-associated hypocapnia. Indeed, a recent study by Ogoh and co-workers [14] reported that dynamic cerebral autoregulation was improved after eight minutes of poikilocapnic hypoxia, whereas it was impaired after eight minutes of isocapnic hypoxia at a F_I_O_2_ of 14%, suggesting that the hyperventilatory response serves to protect the cerebral circulation during acute hypoxia. During more prolonged hypoxia (hours), dynamic cerebral autoregulation has nevertheless been found to be impaired, despite hypocapnia-mediated cerebral vasoconstriction [29]. Therefore, the present findings do not exclude the possibility that the marked hypoxaemia that is observed in critically ill patients with sepsis [4] adversely affects the cerebral circulation during the clinical course of sepsis.

The divergence between the findings relating to the effects of acute isocapnic hypoxia on dynamic autoregulation in the present study and in the study by Ogoh and co-workers [14] is likely related to methodological differences. The latter study used the thigh-cuff deflation technique and subsequent calculation of the rate of regulation for the assessment of dynamic autoregulation [14]. The discrepant findings may thus reflect that the ability of the hypoxic cerebral circulation to respond to an induced reduction in MAP, for example by thigh-cuff deflation, may differ from its ability to dampen the impact of the spontaneous fluctuations in MAP which is assessed by transfer function analysis. In fact, a disassociation between estimates of dynamic cerebral autoregulation upon spontaneous fluctuations and induced manipulations in MAP has been observed in several studies on humans [30,31], which stresses that each approach has its limitations with no universally accepted 'gold standard’. It is possible that the small magnitude and inconsistency of spontaneous pressure oscillations associated with the transfer function analysis approach did not detect subtle imperfections in dynamic cerebral autoregulatory performance during hypoxia in the present study. This might have become apparent if the MAP input signal had been dynamically 'forced’ [31,32]. Future studies may thus benefit from considering transfer function analysis of augmented sinusoidal MAP oscillations forced through various repeated manoeuvers. This, furthermore, has the potential to determine the brain’s differential ability to buffer changes in cerebral perfusion in response to both hyper- and hypotensive challenges, which is important given that hysteresis is a natural characteristic of dynamic autoregulatory responses [33].

LPS infusion was not associated with any changes in COVR to either hyperoxia or hypoxia. By continually monitoring and adjusting P_ET_CO_2_, we successfully managed to maintain PaCO_2_ during the hyperoxic and hypoxic interventions similar to the corresponding normoxic levels at baseline and after LPS, respectively; therefore, our findings are not likely to be biased by the hypoxia-induced hyperventilation which causes hypocapnia-mediated cerebral vasoconstriction and, thus, counteracts hypoxic cerebral vasodilatation [3].

Apart from the limitations relating to the duration of hypoxia and the assessment of dynamic autoregulation, there are a number of methodical limitations relating to the assessment of CBF by TCD that also merit clarification. Firstly, TCD measures the velocity of red blood cells within the insonated vessel, in this instance the middle cerebral artery, and not blood flow as such; secondly, the spatial resolution of TCD-based estimates of cerebral haemodynamic parameters is limited. Changes in MCAv can only be considered reflective of changes in CBF if both the insonation angle and the diameter of the middle cerebral artery are constant. In the present study, the insonation angle was kept constant during the normoxic, hyperoxic and hypoxic interventions by fixing the TCD probe by means of a silicone headband; however, the probe was detached during the four-hour LPS infusion. Although the reported hyperoxia- and hypoxia-induced changes in MCAv are valid, the data at hand, therefore, do not allow for uncritical comparisons of the absolute MCAv values between baseline and LPS. Previous studies have reported a hypocapnia-associated decrease in CBF after LPS administration [10,34] and although hypocapnia was to some extent alleviated in the present study, and the MCAv was not found to be different during normoxia at baseline and after LPS, it cannot be ruled out that a decrease in CBF may have taken place. Even though the diameter of the middle cerebral artery has been documented to be constant during physiological changes in PaCO_2_[18,19] and up to 90 minutes at a F_I_O_2_ of 12% [35], the effects of hyperthermia in this context remain to be clarified. In any event, this is unlikely to influence the reported hyperoxia- and hypoxia- induced changes in MCAv, since the temperature was similar between the interventions at baseline and after LPS, respectively.

With regard to the spatial resolution of TCD, one must bear in mind that the middle cerebral artery delivers oxygenated blood to large regions of the brain, and any TCD-based estimate of cerebral haemodynamic parameters is, thus, to be considered reflective of global, rather than local, cerebrovascular function. It is well-known that there is a noteworthy dissimilarity in vasoreactivity to both oxygen and CO_2_ across the cerebrovasculature [21]; furthermore, by the use of a magnetic resonance imaging-based approach in healthy volunteers, it was recently demonstrated that dynamic cerebral autoregulatory performance is more efficient in white than in grey matter, and more efficient in the cortex than in the cerebellum [36]. Thus, cerebrovascular function in discrete areas of the brain may be affected differently by both changes in MAP, O_2_, CO_2_, hyperthermia and systemic inflammation, changes that may have been rendered undetected by the TCD-based approach used in the present study.

## Conclusions

The present study lends no support to impaired dynamic autoregulation as a contributory factor to sepsis-associated encephalopathy, at least during the very early stages of sepsis as mimicked by use of a LPS infusion in a human-experimental model. Hence, dynamic cerebral autoregulation was improved after LPS and resistant to any adverse effects of acute hypoxia, while COVR to both hyperoxia and hypoxia was unaffected. Further studies on critically ill patients are required to clarify to what extent hypoxia, hyperventilation and the fever response affect dynamic autoregulation during the clinical course of sepsis.

## Key messages

• Dynamic cerebral autoregulation is improved in healthy volunteers after a four-hour lipopolysaccharide (LPS) infusion, a human-experimental model of the very early stages of sepsis.

• Isocapnic hypoxia had no effects on dynamic cerebral autoregulation either before or after LPS, and the cerebral oxygen vasoreactivity to both hyperoxia and hypoxia was found to be intact.

• As the extent of hyperventilation was alleviated by monitoring and adjusting end-tidal CO_2_, the improvement in dynamic cerebral autoregulation was found to be less pronounced than observed during poikilocapnia in former studies.

• Our findings suggest that hyperventilation and the fever response act in synergy to enhance dynamic cerebral autoregulation during the very early stages of sepsis. This may serve to protect the brain from ischaemia and/or blood-brain barrier damage during acute surges in blood pressure.

## Abbreviations

CaO2: Arterial blood content of oxygen; CBF: Cerebral blood flow; COVR: Cerebral oxygen vasoreactivity; FIO2: Inspired fraction of O_2_; IL-6: Interleukin 6; LPS: Lipopolysaccharide; MAP: Mean arterial blood pressure; MAPsp: Spectral power of mean arterial blood pressure; MCAv: Middle cerebral artery blood flow velocity; MCAvsp: Spectral power of middle cerebral artery blood flow velocity; PETCO2: End-tidal CO_2_ tension; TCD: Transcranial Doppler ultrasound; TNF-α: Tumour necrosis factor α.

## Competing interests

The authors declare that they have no competing interests.

## Authors’ contributions

RMGB was the principal investigator, and conceived and designed the research, conducted the study, acquired, analysed and interpreted the data, performed statistical analyses, drafted the manuscript and handled funding. RRP conducted the study, acquired, analysed and interpreted the data and performed statistical analyses. KAE conducted the study, acquired, analysed and interpreted the data. CBC conducted the study. DMB analysed and interpreted the data. NHHR analysed and interpreted the data and handled supervision. KM conceived and designed the research, analysed and interpreted the data and handled funding and supervision. All authors made critical revisions and read and approved the final manuscript.

## Supplementary Material

Additional file 1 **Title: Symptom scores.** Description: Lipopolysaccharide (LPS) was administered as a continuous four-hour intravenous infusion from 0 to 4 hours at an infusion rate of 0.5 ng.kg^-1^.hour^-1^ in healthy volunteers (n = 10). Volunteer symptoms were evaluated at these time points by means of a modified visual analogue scale, in which each symptom was rated from 1 (implying that the symptom was not present at all) to 10 (implying that the symptom was the worst imaginable). Individual symptom scores were added together to yield a total symptom score with a minimal value of 6 and a maximal value of 60. Data are presented as median (interquartile range). #P-value for LPS x time interaction (linear mixed model); different from baseline (after Tukey-Kramer adjustment for multiple comparisons), *P<0.05, **P<0.01, ***P<0.001 and ****P<0.0001. Click here for file

Additional file 2 **Title: Transfer function analysis.** Description: Transfer function analysis between spontaneous oscillations in mean arterial blood pressure (MAP) and middle cerebral artery blood flow velocity (MCAv) performed during three interventions, normoxia (F_I_O_2_ = 21%), hyperoxia (F_I_O_2_ = 40%) and hypoxia (F_I_O_2_ = 12%), at baseline, and after a four-hour lipopolysaccharide (LPS) infusion at an infusion rate of 0.5 ng.kg^-1^ .hour^-1^ in healthy volunteers (n = 10). Values for the spectral power of MAP (MAP_sp_) and MCAv (MCAv_sp_), as well as transfer gain, normalised gain, the MAP-to-MCAv phase difference, and coherence are presented for the very low (VLF, 0.02 to 0.07 Hz), low (LF, 0.07 to 0.20 Hz), and high (HF, 0.20 to 0.30 Hz) frequency ranges, respectively. Data are presented as median (interquartile range). Different from normoxia in the same condition (baseline/LPS), *P <0.05. Different from the same intervention (normoxia/hyperoxia/hypoxia) at baseline, † *P* <0.05, †† *P* <0.01. Click here for file
