# Supplementary material for: Lipopolysaccharide infusion enhances dynamic cerebral autoregulation without affecting cerebral oxygen vasoreactivity in healthy volunteers
Source: Crit Care. 2013 Oct 16;17(5):R238. doi: 10.1186/cc13062 (PMC4057209; doi:10.1186/cc13062)
Supplement: Additional file 1 — Title: Symptom scores. Description: Lipopolysaccharide (LPS) was administered as a continuous four-hour intravenous infusion from 0 to 4 hours at an infusion rate of 0.5 ng.kg-1.hour-1 in healthy volunteers (n = 10). Volunteer symptoms were evaluated at these time points by means of a modified visual analogue scale, in which each symptom was rated from 1 (implying that the symptom was not present at all) to 10 (implying that the symptom was the worst imaginable). Individual symptom scores were added together to yield a total symptom score with a minimal value of 6 and a maximal value of 60. Data are presented as median (interquartile range). #P-value for LPS x time interaction (linear mixed model); different from baseline (after Tukey-Kramer adjustment for multiple comparisons), *P<0.05, **P<0.01, ***P<0.001 and ****P<0.0001. [file cc13062-S1.pdf]

	0h	1h	2h	3h	4h	6h	$p^{\#}$
**Malaise**	2 (1–3)	2 (1–2)	3 (2–3)	5**** (3–5)	5**** (4–5)	1 (1–2)	<0.0001
**Shivering**	1 (1–1)	1 (1–1)	2 (1–2)	6**** (3–6)	4*** (3–6)	1 (1–1)	<0.0001
**Nausea**	1 (1—1)	1 (1–1)	1 (1–1)	1 (1–2)	2 (1–2)	1 (1–2)	0.1002
**Headache**	1 (1–1)	2 (1–2)	2* (2–4)	3** (2–5)	4**** (2–6)	1 (1–1)	<0.0001
**Myalgia**	1 (1–1)	1 (1–2)	2 (1–2)	2** (1–3)	2** (2–3)	1 (1–1)	0.0008
**Dizziness**	1 (1–2)	1 (1–2)	2 (1–3)	2 (1–4)	2 (1–4)	1 (1–2)	0.0054
**Total**	8 (6–9)	9 (8–9)	12 (10–15)	17*** (14–23)	19**** (15–22)	7 (6–8)	<0.0001

Additional file 1. Effects of a lipopolysaccharide (LPS) infusion on symptom scores in healthy volunteers (n = 10). LPS was administered as a continuous 4-hour intravenous infusion from 0 to 4h at an infusion rate of 0.5 ng kg^{-1} $hour^{-1}$. Volunteer symptoms were evaluated at these time points by means of a modified visual analogue scale, in which each symptom was rated from 1 (implying that the symptom was not present at all) to 10 (implying that the symptom was the worst imaginable). Individual symptom scores were added together to yield a total symptom score with a minimal value of 6 and a maximal value of 60. Data are presented as median (IQR). # p-value for LPS x time interaction (linear mixed model); different from baseline (after Tukey-Kramer adjustment for multiple comparisons), * p < 0.05, ** p < 0.01, *** p < 0.001, and **** p < 0.0001.
