# Supplementary material for: Lipopolysaccharide infusion enhances dynamic cerebral autoregulation without affecting cerebral oxygen vasoreactivity in healthy volunteers
Source: Crit Care. 2013 Oct 16;17(5):R238. doi: 10.1186/cc13062 (PMC4057209; doi:10.1186/cc13062)
Supplement: Additional file 2 — Title: Transfer function analysis. Description: Transfer function analysis between spontaneous oscillations in mean arterial blood pressure (MAP) and middle cerebral artery blood flow velocity (MCAv) performed during three interventions, normoxia (FIO2 = 21%), hyperoxia (FIO2 = 40%) and hypoxia (FIO2 = 12%), at baseline, and after a four-hour lipopolysaccharide (LPS) infusion at an infusion rate of 0.5 ng.kg-1 .hour-1 in healthy volunteers (n = 10). Values for the spectral power of MAP (MAPsp) and MCAv (MCAvsp), as well as transfer gain, normalised gain, the MAP-to-MCAv phase difference, and coherence are presented for the very low (VLF, 0.02 to 0.07 Hz), low (LF, 0.07 to 0.20 Hz), and high (HF, 0.20 to 0.30 Hz) frequency ranges, respectively. Data are presented as median (interquartile range). Different from normoxia in the same condition (baseline/LPS), *P <0.05. Different from the same intervention (normoxia/hyperoxia/hypoxia) at baseline, † P <0.05, †† P <0.01. [file cc13062-S2.pdf]

	Baseline			LPS		
	Normoxia	Hyperoxia	Hypoxia	Normoxia	Hyperoxia	Hypoxia
**VLF**						
**MAP_{sp}** ($mmHg^2$	16.1 (12.5–23.4)	16.1 (11.9–25.8)	18.3 (15.2–23.2)	6.8† (5.2–8.3)	7.9† (6.7–10.3)	10.0† (6.2–12.8)
**$MCAv_{sp}$** (cm^2 sec^{-2})	31.8 (17.7–54.1)	25.3 (10.9–35.1)	28.3 (14.1–54.2)	10.7† (5.5–18.3)	14.7 (11.3–22.1)	15.8 (9.6–25.0)
**Gain** (cm $mmHg^{-1}$ sec^{-1})	0.83 (0.65–1.72)	0.67 (0.52–1.04)	0.86 (0.46–1.38)	0.66 (0.50–0.83)	0.64 (0.59–0.78)	0.85 (0.71–0.91)
**Normalised gain** (units)	1.17 (0.80–1.51)	0.92* (0.75–1.25)	0.84 (0.75–1.25)	0.78 (0.57–1.15)	0.75 (0.71–0.90)	0.89 (0.67–1.02)
**Phase** (radians)	0.91 (0.32–1.50)	1.21 (1.00–1.58)	1.05 (0.46–1.38)	0.82 (0.34–1.42)	1.67 (1.41–2.06)	0.71 (0.25–1.86)
**Coherence** (units)	0.65 (0.58–0.78)	0.59 (0.56–0.73)	0.63 (0.59–0.84)	0.55 (0.44–0.64)	0.51 (0.44–0.61)	0.66 (0.61–0.78)
**LF**						
**MAP_{sp}** ($mmHg^2$)	4.5 (3.5–6.3)	4.5 (4.2–6.3)	9.0 (4.7–13.8)	3.6 (1.9–9.3)	8.0 (4.6–10.3)	8.1 (1.9–10.7)
**$MCAv_{sp}$** (cm^2 sec^{-2})	7.7 (5.5–10.0)	7.6 (5.3–11.4)	12.6 (7.6–18.7)	5.6 (1.8–14.2)	11.6 (8.8–13.5)	11.4 (4.4–21.0)
**Gain** (cm $mmHg^{-1}$ sec^{-1})	1.24 (1.04–1.36)	1.31 (1.06–1.38)	1.27 (1.18–1.34)	1.17 (1.00–1.24)	1.17 (0.99–1.28)	1.24 (1.04–1.32)
**Normalised gain** (units)	1.43 (1.36–1.49)	1.48 (1.36–1.59)	1.32 (1.26–1.50)	1.27 (1.13–1.40)	1.34 (1.24–1.49)	1.21 (1.09–1.40)
**Phase** (radians)	0.64 (0.49–0.76)	0.70 (0.52–0.79)	0.49 (0.45–0.60)	0.80†† (0.77–0.83)	0.82†† (0.78–0.98)	0.87 (0.69–1.01)
**Coherence** (units)	0.87 (0.82–0.88)	0.89 (0.86–0.93)	0.91 (0.89–0.93)	0.76 (0.70–0.84)	0.86 (0.83–0.90)	0.87 (0.66–0.94)
**HF**						
**MAP_{sp}** ($mmHg^2$)	0.9 (0.6–3.0)	1.2 (0.7–2.5)	2.2** (1.6–4.1)	1.5 (1.0–2.2)	2.8 (1.3–4.5)	3.0 (2.2–6.9)
**$MCAv_{sp}$** (cm^2 sec^{-2})	2.5 (1.4–3.2)	3.1 (1.4–4.4)	4.3 (2.4–7.6)	4.3 (2.5–7.3)	7.1 (3.9–10.3)	6.8 (4.0–8.0)
**Gain** (cm $mmHg^{-1}$ sec^{-1})	1.43 (1.25–1.52)	1.46 (1.17–1.60)	1.33 (1.27–1.54)	1.52 (1.42–1.79)	1.46 (1.19–1.73)	1.43 (1.12–1.61)
**Normalised gain** (units)	1.52 (1.49-1.67)	1.65 (1.51-1.79)	1.56 (1.35-1.59)	1.85 (1.46-1.92)	1.68 (1.43-1.82)	1.42 (1.23-1.78)
**Phase** (radians)	-0.03 ([-0.11]–0.11)	0.04 ([-0.15]–0.23)	0.12 ([-0.01]–0.29)	0.43†† (0.36–0.56)	0.38†† (0.25–0.50)	0.44† (0.36–0.64)
**Coherence** (units)	0.89 (0.84–0.92)	0.90 (0.89–0.92)	0.92 (0.82–0.95)	0.91 (0.85–0.92)	0.88 (0.82–0.92)	0.92 (0.86–0.94)

Additional file 2. Transfer function analysis during inspiratory hyperoxia and hypoxia, before and after lipopolysaccharide (LPS) infusion in healthy volunteers (n = 10). Transfer function analysis between spontaneous oscillations in mean arterial blood pressure (MAP) and middle cerebral artery blood flow velocity (MCAv) performed during three interventions, normoxia (F_IO_2 = 21 %), hyperoxia (F_IO_2 = 40 %) and hypoxia (F_IO_2 = 12 %), at baseline, and after a 4-hour LPS infusion at an infusion rate of 0.5 ng kg^{-1} $hour^{-1}$. Values for the spectral power of MAP (MAP_{sp}) and MCAv ($MCAv_{sp}$), as well as transfer gain, normalised gain, the MAP-to-MCAv phase difference, and coherence are presented for the very low (VLF, 0.02–0.07 Hz), low (LF, 0.07–0.20 Hz), and high (LF, 0.20–0.30 Hz) frequency ranges, respectively. Data are presented as median (IQR). Different from normoxia in the same condition (baseline/LPS), * $p < 0.05$. Different from the same intervention (normoxia/hyperoxia/hypoxia) at baseline, † $p < 0.05$, †† $p < 0.01$.
